# Antimicrobial Efficacy of Indolicidin Against Multi-Drug Resistant Enteroaggregative *Escherichia coli* in a *Galleria mellonella* Model

**DOI:** 10.3389/fmicb.2019.02723

**Published:** 2019-11-29

**Authors:** Jess Vergis, Satyaveer Singh Malik, Richa Pathak, Manesh Kumar, Sunitha Ramanjaneya, Nitin Vasantrao Kurkure, Sukhadeo Baliram Barbuddhe, Deepak Bhiwa Rawool

**Affiliations:** ^1^Division of Veterinary Public Health, ICAR-Indian Veterinary Research Institute, Bareilly, India; ^2^Department of Veterinary Pathology, Nagpur Veterinary College, Nagpur, India; ^3^ICAR-National Research Centre on Meat, Hyderabad, India

**Keywords:** antimicrobial peptide, enteroaggregative *Escherichia coli*, *Galleria mellonella*, Indolicidin, multi-drug resistance

## Abstract

Antimicrobial resistance against enteroaggregative *Escherichia coli* (EAEC), an emerging food-borne pathogen, has been observed in an increasing trend recently. In the recent wake of antimicrobial resistance, alternate strategies especially, cationic antimicrobial peptides (AMPs) have attracted considerable attention to source antimicrobial technology solutions. This study evaluated the *in vitro* antimicrobial efficacy of Indolicidin against multi-drug resistant enteroaggregative *Escherichia coli* (MDR-EAEC) strains and further to assess its *in vivo* antimicrobial efficacy in *Galleria mellonella* larval model. The minimum inhibitory concentration (MIC; 32 μM) and minimum bactericidal concentration (MBC; 64 μM) of Indolicidin against MDR-EAEC was determined by micro broth dilution method. Indolicidin was also tested for its stability (high-end temperatures, physiological concentration of salts and proteases); safety (sheep RBCs; HEp-2 and RAW 264.7 cell lines); effect on beneficial microflora (*Lactobacillus rhamnosus* and *Lactobacillus acidophilus*) and its mode of action (flow cytometry; nitrocefin and ONPG uptake). *In vitro* time-kill kinetic assay of MDR-EAEC treated with Indolicidin was performed. Further, survival rate, MDR-EAEC count, melanization rate, hemocyte enumeration, cytotoxicity assay and histopathological examination were carried out in *G. mellonella* model to assess *in vivo* antimicrobial efficacy of Indolicidin against MDR-EAEC strains. Indolicidin was tested stable at high temperatures (70°C; 90°C), physiological concentration of cationic salts (NaCl; MgCl_2_) and proteases, except for trypsin and tested safe with sheep RBCs and cell lines (RAW 264.7; HEp-2) at MIC (1X and 2X); the beneficial flora was not inhibited. Indolicidin exhibited outer membrane permeabilization in a concentration- and time-dependent manner. *In vitro* time-kill assay revealed concentration-cum-time dependent clearance of MDR-EAEC in Indolicidin-treated groups at 120 min, while, in *G. mellonella*, the infected group treated with Indolicidin revealed an increased survival rate, immunomodulatory effect, reduced MDR-EAEC counts and were tested safe to the larval cells which was concurred histopathologically. To conclude, the results suggests Indolicidin as an effective antimicrobial candidate against MDR-EAEC and we recommend its further investigation in appropriate animal models (mice/piglets) before its application in the target host.

## Introduction

Cationic antimicrobial peptides (AMPs) have emerged as an attractive target to source new antimicrobial technology solutions. AMPs are evolutionarily conserved molecules found in organisms ranging from prokaryotes to humans that have been heralded as promising alternatives to the currently available antibiotics (Yazici et al., 2018; Haney et al., 2019). Employing long chain amino acid sequences increase the output cost of peptides and thereby the cost of research; hence, synthetic short-chain cationic peptides with potential antimicrobial activity have been attempted (Anunthawan et al., 2015). In particular, Indolicidin, a tridecapeptide isolated from the cytoplasmic granules of bovine neutrophils, was reported to exhibit membrane permeabilization effects and antimicrobial activity against Gram-negative and -positive bacteria, fungi, HIV-1 virus and protozoa (Falla et al., 1996; Rokitskaya et al., 2011). However, barring a few systematic studies, the use of AMPs against multi-drug resistant pathogens such as enteroaggregative *Escherichia coli* (EAEC), remains unrevealed (Reyes-Cortes et al., 2016; Pollini et al., 2017).

Enteroaggregative *Escherichia coli*, an emerging food-borne pathogen, are implicated in endemic as well as epidemic diarrheal episodes. EAEC is considered to be heterogeneous in nature and its pathogenicity is described in three distinct stages: an initial adherence to the intestinal mucosal surface, biofilm formation and induction of inflammatory response resulting in the toxin release (Lima et al., 2018). Multi-drug resistance toward the antibiotics of first-line empirical therapy (fluoroquinolones and β-lactams) has been evident globally among the EAEC isolates (Lima et al., 2018; Guiral et al., 2019). Therefore, approaches such as antibiotic stewardship, public health education, changing social norms and novel diagnostics and therapeutics are initiated in most of the developing countries, including India (Laxminarayan and Chaudhury, 2016).

*In vivo* clinical manifestations of EAEC have been established in piglet and murine models for evaluation of novel therapeutics which possess ethical, budgetary and logistical hurdles (Philipson et al., 2013; Kumar et al., 2016). *Galleria mellonella* (Lepidoptera: Pyralidae), which is easier and cheaper to procure, establish and maintain, has been introduced as an alternative model to study the microbial infections, including EAEC (Jønsson et al., 2017). The short life span and ability of larvae to mimic the human host while investigating the clinically relevant human pathogens at 37°C suites them an ideal *in vivo* model for high throughput studies (Tsai et al., 2016; Wojda, 2017). The objective of the present study was to evaluate the *in vitro* antimicrobial efficacy of Indolicidin against multi-drug resistant enteroaggregative *Escherichia coli* (MDR-EAEC) strains and further to assess their *in vivo* efficacy in *G. mellonella* model.

## Materials and Methods

### Bacterial Strains, Media, and Culture Conditions

The typical EAEC strains, isolated from the fecal samples of human infants with GenBank accession numbers, KY941936.1 (MDR 1); KY941937.1 (MDR 2); and KY941938.1 (MDR 3), available at Division of Veterinary Public Health, Indian Veterinary Research Institute, Bareilly were re-validated as described earlier (Vijay et al., 2015) and tested for antibiotic susceptibility (Clinical and Laboratory Standards Institute [CLSI], 2018). *E. coli* ATCC 25922 used as quality control strain was provided by the Department of Veterinary Public Health, College of Veterinary and Animal Sciences, Pookode, India. These bacterial strains were grown on nutrient agar medium at 37°C.

### Antimicrobial Peptide

Indolicidin (Supplementary Table S1) retrieved from BaAMPs (Di Luca et al., 2015) was synthesized commercially (Shanghai Science Peptide Biological Technology, China), resuspended in PBS (final stock concentration of 10 mg/mL) and stored at −20°C until further use.

### Characterization of Indolicidin

Indolicidin was characterized for minimum inhibitory concentration (MIC), minimum bactericidal concentration (MBC) (Table 1), *in vitro* stability (temperature, proteases, physiological concentration of salts) assays, *in vitro* safety (hemolysis and cytotoxicity) assays (Supplementary Files S1–S3 and Supplementary Files S3–S6). The membrane permeabilization effect of Indolicidin on MDR-EAEC strains (*n* = 3) was assessed by flow cytometry while, the outer and inner membrane permeability of MDR-EAEC isolates treated with MIC (1X and 2X) values of Indolicidin was carried out based on the nitrocefin activity as well as release of cytoplasmic β-galactosidase activity, respectively (Supplementary Files S4–S6). Further, Indolicidin was evaluated for its antibacterial effect against commensal gut flora (*Lactobacillus rhamnosus* MTCC 1408 and *Lactobacillus acidophilus* MTCC 10307) (Supplementary File S7).

**TABLE 1:** MIC and MBC observed for Indolicidin against MDR-EAEC isolates.

**NCBI GenBank accession no.**	**Indolicidin**	
	**MIC (μM)**	**MBC (μM)**
KY941936.1 (MDR 1)	32.0	64.0
KY941937.1 (MDR 2)	32.0	64.0
KY941938.1 (MDR 3)	32.0	32.0

### *In vitro* Dose- and Time-Dependent Growth Kinetics of MDR-EAEC With Indolicidin

The *in vitro* growth kinetics of MDR-EAEC isolates was evaluated by incubating the log-phase bacterial cultures of each MDR-EAEC isolate (ca. 1 × 10^7^ CFU/mL) in CA-MH broth with MIC (1X and 2X) concentrations of Indolicidin, in triplicates (Supplementary File S8). The desired bacterial numbers for each MDR-EAEC isolate and Indolicidin were suspended in CA-MH broth as follows: Group I, 10^7^ CFU of MDR-EAEC (50 μL) with 1X MIC Indolicidin (50 μL); Group II, 10^7^ CFU of MDR- EAEC (50 μL) with MBC Indolicidin (50 μL); Group III, 10^7^ CFU of MDR- EAEC (50 μL) with Meropenem (10 μg/ml; 50 μL); Group IV, 10^7^ CFU of MDR- EAEC (50 μL) with CA-MH broth (50 μL) and Group V, 10^7^ CFU of MDR- EAEC (50 μL) with 4X MIC Indolicidin (50 μL). To enumerate the antibacterial effect of Indolicidin on MDR-EAEC isolates, an aliquot at 10 μL from all the five groups were drawn at 0, 30, 60, 90, 120, 150, 180 min, 24, 48, and 72 h. These aliquots were serially diluted 10-fold in normal saline solution (prepared using 0.90% of sodium chloride, HiMedia Laboratories); the last three dilutions placed on EMB agar plates containing 100 μg of ampicillin (Miles et al., 1938) were counted after 24 h of incubation at 37°C, and the bacterial counts were expressed as log_10_CFU/mL.

### *In vivo* Assays Using *G. mellonella* Model

*In vivo* assays were performed using the final instar of *G. mellonella* larvae that were stored in wood shavings at 15°C in the dark prior to the experiment (Morgan et al., 2014). The larvae were kept in a germ-free environment and were provided with *ad libitum* food during the course of experiment. Initially, LD_50_ dose of each MDR-EAEC strains was determined in *G. mellonella* larvae, and the validated LD_50_ dose was used further in the *in vivo* studies to evaluate the antibacterial effect of Indolicidin (Supplementary File S9) by injection with a Hamilton syringe (26 gage) via the last right pro-leg.

#### *In vivo* Evaluation of Antimicrobial Efficacy of Indolicidin Against MDR-EAEC Strains

*Galleria mellonella* larvae (*n* = 40 larvae per group) were grouped as follows: Group I (infected group), Groups II and III (infection + treatment groups), Group IV (PBS control), and Group V (AMP control). Larvae from groups I to III were infected with cocktail of MDR-EAEC strains (LD_50_ dose; 10 μL); groups II and III were administered 3 h post-infection (pi) with MIC dose (10 μL) of Indolicidin and Meropenem, respectively; Group IV were injected with sterile PBS whereas, group V was administered with MIC dose of Indolicidin. The larvae were observed for their melanization (Supplementary File S10), MDR-EAEC counts (Supplementary File S11) and death, at an interval of 6 h upto 24 h, followed by 24 h interval till 120 h pi to determine the survival rate.

#### Enumeration of Hemocytes

The hemocyte density of *G. mellonella* (*n* = 3 larvae per group) at an interval of 6 h pi upto 24 h, followed by 24 h interval till 96 h pi were quantified as described (Gibreel and Upton, 2013). No attempt was made to discriminate between the different hemocyte subtypes.

#### Lactate Dehydrogenase (LDH) Cytotoxicity Assay

*Galleria mellonella* (*n* = 3 larvae per group) were analyzed for the production of LDH, as a marker of cell damage, at an interval of 6 h pi for 24 h, followed by 24 h interval till 96 h pi using QuantiChrom LDH cytotoxicity assay kit (Supplementary File S12), according to the manufacturer’s instructions (Wand et al., 2013).

#### Histopathological Examination

The larvae at each time point were subjected to histopathological examination (Supplementary File S13) to study the tissue level changes (Perdoni et al., 2014). The microscopic visualization was performed (Leica Microscope DMLB) and the image acquisition was carried out (NanoZoomer-XR C12000, Hamamatsu Photonics, Japan).

### Statistical Analysis

All the experiments were repeated individually and independently thrice and the data obtained is reflected as mean ± standard deviation for each assay. GraphPad Prism 8.2.1 software (GraphPad Software Inc., San Diego, CA, United States) was used for statistical analysis. A one-way analysis of variance (ANOVA) with Bonferroni multiple comparison post-test was used to compare the differences between cytotoxicity of control and AMP-treated cell lines. The association of AMPs on commensal gut flora was measured by paired two-tailed “t” test. A two-way (repeated measures) ANOVA with Bonferroni multiple comparison post-test was used to compare the differences between control and AMP-treated tests for the *in vitro* and *in vivo* time-dependent antimicrobial assays. *In vivo G. mellonella* larval survival curves were analyzed by log rank (Mantel-Cox) test and log rank test for trends while, the LD_50_ of the MDR-EAEC isolates were determined by probit-regression model.

## Results

The three typical MDR-EAEC isolates included in the study were resistant to four or more classes of antibiotics and were ESBL-producers (Supplementary Table S2).

### *In vitro* Killing Kinetics of MDR-EAEC With Indolicidin

**FIGURE 1:**
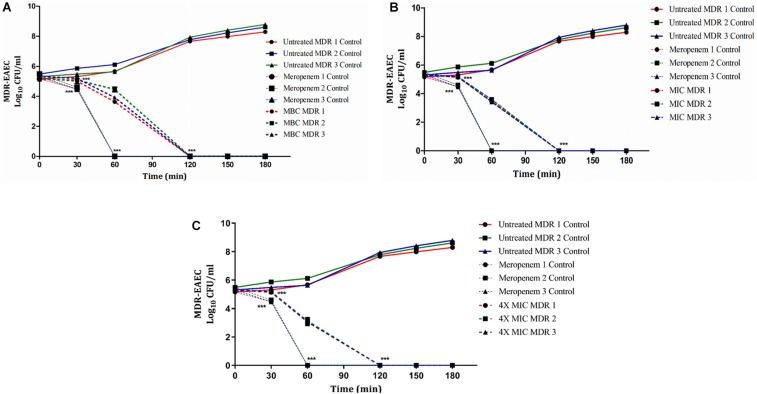
Dose- and time-dependent killing kinetics of MDR-EAEC isolates co-cultured with Indolicidin at different time intervals. Three MDR-EAEC isolates were co-cultured with: MBC of Indolicidin **(A)**; MIC of Indolicidin **(B)**; 4X MIC of Indolicidin **(C)** in CA-MH broth at 37°C under static conditions. Simultaneously, respective controls of MDR-EAEC isolates (untreated and meropenem-treated) were incubated in CA-MH broth. Data expressed as the mean ± standard deviation (log_10_CFU/mL) of three independent experiments. Error bars are so close to display. ^∗∗∗^*P* < 0.001.

In groups I, II, and V, the antimicrobial effect of Indolicidin was highly significant (*P* < 0.001) at 120 min of co-incubation (Figure 1). However, though the antimicrobial effect of Indolicidin at 4X MIC, MBC, MIC was highly significant at 120 min, the reduction in bacterial count was slightly lower in group V (mean 2.60 log reduction) as compared to group I (mean 2.40 log reduction) and group II (mean 1.85 log reduction). Nevertheless, group III exhibited highly significant (*P* < 0.001) reduction at 60 min of co-incubation. After 120 min of co-incubation, none of the MDR-EAEC isolates in groups I, II, III, and V exhibited any visible growth whereas, in group IV, all the MDR-EAEC isolates exhibited an increasing growth pattern at 30, 60, 90, 120, 150, and 180 min of incubation (Figure 1). Since the MIC value of Indolicidin was observed to be equally effective when compared with the MBC and 4X MIC to inhibit the growth of all the three MDR-EAEC strains (Figure 1), further studies employed the use of 1X MIC levels to investigate the *in vivo* antimicrobial activity.

### Determination of LD_50_ of MDR-EAEC Strains in *G. mellonella* Larvae

Inoculation of *G. mellonella* with MDR-EAEC strains resulted larval killing in a bacterial concentration-dependent manner (Supplementary File S13). Based on the survival study, 10^6^ CFU/larvae was determined as LD_50_ dose in *G. mellonella* larvae.

### *In vivo* Evaluation of Antimicrobial Efficacy of AMPs Against MDR-EAEC

**FIGURE 2:**
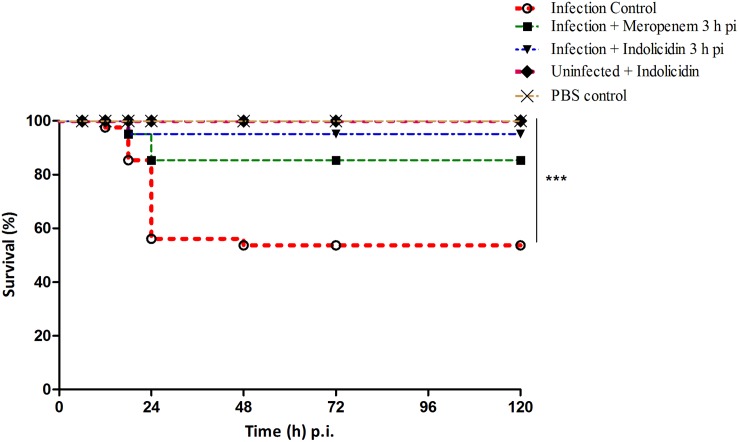
Survival of *G. mellonella* larvae infected with LD_50_ dose of MDR-EAEC strains (10^6^ CFU) and treated with MIC dose of Indolicidin 3 h post-infection. MDR-EAEC induced infection (10 μL) was treated with MIC dose of Indolicidin (10 μL), keeping respective controls (infected, infected with meropenem-treatment, AMP control, PBS control). Data expressed as the mean of three independent experiments. Survival curves were plotted using the Kaplan–Meier method and statistical analysis were performed using the log-rank test for multiple comparisons (GraphPad Software, San Diego, CA, United States). ^∗∗∗^*P* < 0.001.

In infected group of larvae (Group I), a survival rate of 52.50% was observed while, the meropenem treated group (Group III) exhibited a survival rate of 85% upto 120 h pi (Figure 2). An enhanced survival rate of 95% was exhibited by the Indolicidin treated infected larval group (Group II) that corresponded to a highly significant logrank Mantel–Cox test (*P* < 0.001) and logrank test for trend (*P* < 0.01) (Figure 2). All the uninfected larvae of PBS control group (Group IV) as well as those administered with Indolicidin (Group V) were found healthy and 100% survival rate was observed upto 120 h pi (Figure 2).

### Melanization Assay

**FIGURE 3:**
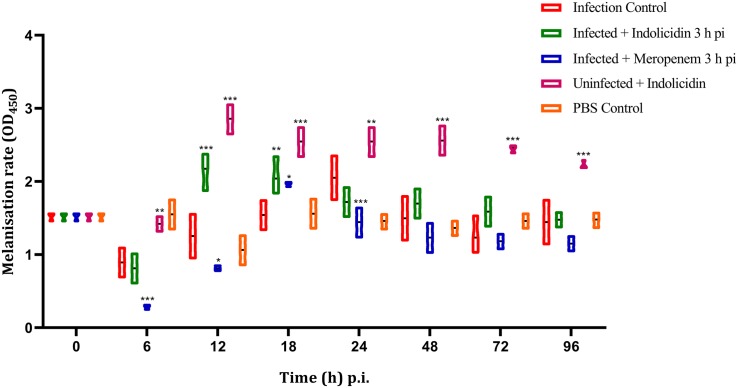
Melanization rate of *G. mellonella* larvae infected with LD_50_ dose of MDR-EAEC strains (10^6^ CFU) and treated with MIC dose of Indolicidin 3 h post-infection. MDR-EAEC induced infection (10 μL) was treated with MIC of Indolicidin (10 μL), keeping respective controls (infected, infected with meropenem-treatment, AMP control, PBS control). Data expressed as the mean ± standard deviation of three independent experiments by absorbance monitored at 450 nm. Statistical analysis of melanization rate was performed using the two-way (repeated measures) ANOVA with Bonferroni multiple comparison post-test. ^∗∗∗^*P* < 0.001, ^∗∗^*P* < 0.01, ^∗^*P* < 0.05.

The melanization of larvae in infected group (Group I) was lower at 6 h pi, thereafter increased gradually, reached its peak at 24 h pi; the melanization was found to decline at 48 h pi (Figure 3). However, in meropenem treated group (Group III), the melanization was lower at 6 h pi, reached its peak at 18 h pi and thereafter gradually declined in a highly significant (*P* < 0.001) manner (Figure 3). In Group II (Indolicidin treatment), melanization was found to increase since 12–18 h pi, however, at later time point, a gradual decline in melanization was observed (Figure 3). In uninfected larval group treated with Indolicidin (group V), a slight increase in intensity of melanization was observed at 6 h pi, thereafter, from 12 to 96 h pi, the increased intensity of melanization was retained (Figure 3).

### Enumeration of MDR-EAEC Counts

**FIGURE 4:**
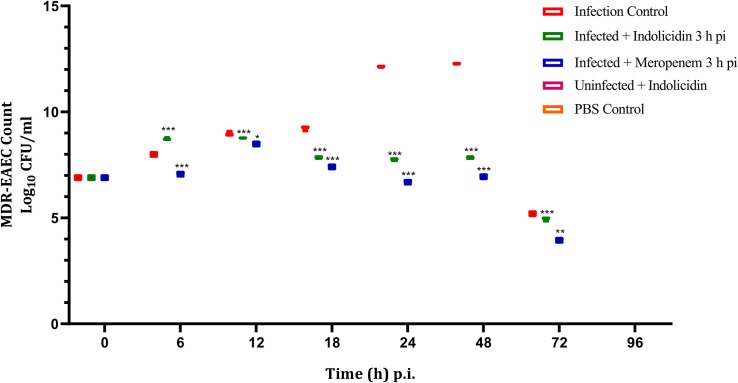
MDR-EAEC counts of *G. mellonella* larvae infected with LD_50_ dose of MDR-EAEC strains (10^6^ CFU) and treated with MIC dose of Indolicidin 3 h post-infection. MDR-EAEC induced infection (10 μL) was treated with MIC of Indolicidin (10 μL), keeping respective controls (infected, infected with meropenem-treatment, AMP control, PBS control). Data expressed as the mean ± standard deviation (log_10_CFU/mL of hemolymph) of three independent experiments on EMB agar plates supplemented with ampicillin (100 μg/plate). Statistical analysis of MDR-EAEC counts was performed using the two-way (repeated measures) ANOVA with Bonferroni multiple comparison post-test. ^∗∗∗^*P* < 0.001, ^∗∗^*P* < 0.01, ^∗^*P* < 0.05.

The infected larval group treated with Indolicidin (Group II) revealed a significant reduction (*P* < 0.001) in MDR-EAEC counts at 24 h pi as compared to the infected group (Group I). Further, significant reduction (*P* < 0.001) in MDR-EAEC counts was observed in group II at 48 and 72 h pi (Figure 4). Further, in all the uninfected larval groups, MDR-EAEC were not detected from the hemolymph of larvae till 96 h pi (Figure 4).

### Enumeration of Hemocytes

**FIGURE 5:**
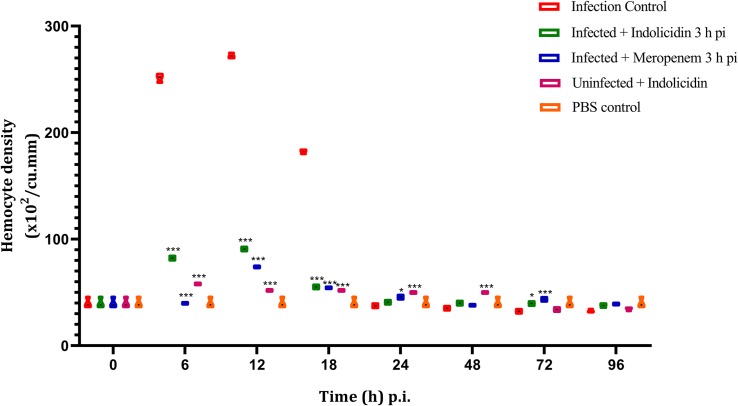
Hemocyte density of *G. mellonella* larvae infected with LD_50_ dose of MDR-EAEC strains (10^6^ CFU) and treated with MIC dose of Indolicidin 3 h post-infection. MDR-EAEC induced infection (10 μL) was treated with MIC of Indolicidin(10 μL), keeping respective controls (infected, infected with meropenem-treatment, AMP control, PBS control). Data expressed as the mean ± standard deviation (cells/mL of hemolymph) of three independent experiments. Statistical analysis of hemocyte density was performed using the two-way (repeated measures) ANOVA with Bonferroni multiple comparison post-test. ^∗∗∗^*P* < 0.001, ^∗^*P* < 0.05.

Irrespective of the infected as well as treatment groups of larvae, the hemocyte density was found to increase significantly (*P* < 0.001) at 6 h pi, reached its peak by 12 h and thereafter, declined in significant (*P* < 0.001) manner (Figure 5); however, from 72 to 96 h pi, significant difference in hemocyte density was not observed (*P* > 0.05) between any of the larval groups (Figure 5).

### LDH Cytotoxicity Assay

**FIGURE 6:**
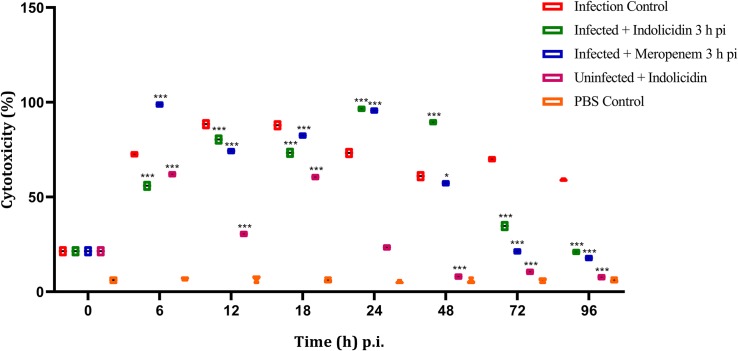
LDH cytotoxicity assay of *G. mellonella* larvae infected with LD_50_ dose of MDR-EAEC strains (10^6^ CFU) and treated with MIC dose of Indolicidin 3 h post-infection. MDR-EAEC induced infection (10 μL) was treated with MIC of Indolicidin (10 μL), keeping respective controls (infected, infected with meropenem-treatment, AMP control, PBS control). Data expressed as the cytotoxicity (%) of larval hemolymph of three independent experiments. Statistical analysis of LDH cytotoxicity assay was performed using the two-way (repeated measures) ANOVA with Bonferroni multiple comparison post-test. ^∗∗∗^*P* < 0.001, ^∗^*P* < 0.05.

In group I, the LDH cytotoxicity was observed to increase in a highly significant manner (*P* < 0.001) at 6 h pi, reached its peak at 12–18 h pi and thereafter retained cytotoxicity upto 96 h pi (Figure 6). In group II, a significant (*P* < 0.001) increase in cytotoxicity was observed at 6 h pi and thereafter, the cytotoxicity remained elevated till 48 h pi, later, a progressive decline in the cytotoxicity was noticed (Figure 6). However, in group III, a highly significant (*P* < 0.001) cytotoxicity was observed from 6 to 48 h pi and started declining thereafter upto 96 pi (Figure 6). In group V, a significant increase in cytotoxicity was observed from 6 to 18 h pi, thereafter, it declined progressively (Figure 6).

### Histopathological Examination

All the larval groups, except for group I (infection control), at 6and 12 h pi did not reveal any alteration in the tissue structure while, at 12–18 h pi, scanty distribution of hemocytes with no noticeable aggregates or melanization was observed. However, in group I, at 12–18 h pi, distribution of hemocytes was more pronounced in sub-cuticular area exhibiting phagocytic reaction of bacteria with an evidence of melanization and bacterial load around the tubular organs. At 18 h pi, H&E stained cross-sections of group I revealed clusters of hemocytes in the sub-cuticular area exhibiting phagocytosis of bacteria (finely stippled blue dots) with an evidence of melanization. Besides, tubular organs were surrounded with load of bacteria. In contrast, larval cross-section of group II and IV and V looked apparently healthy with individually distributed scanty hemocytes exhibiting no noticeable aggregates or melanization (Supplementary Figure S7).

Collectively, the infected control larvae exhibited increased pathological abnormalities at 24 and 48 h pi, which later at 72 h pi, was progressively declined whereas, mild to moderate histopathological changes were evident in Indolicidin treated groups at 24 h of pi, which later at 48 and 72 h pi declined progressively. Surprisingly, no abnormal pathological changes could be observed in uninfected control group and PBS control group.

## Discussion

Multi-drug resistance developed due to “antibiotic selection pressure” makes the pathogens impervious to a varied class of antibiotics, specifically recommended for empirical therapy (Davies and Davies, 2010). With limited availability of antibiotics as well as similarities in their mode of action, intensive research is directed toward the identification of novel and non-conventional therapeutics (Batoni et al., 2016; de la Fuente-Núñez et al., 2016; Haney et al., 2019). Recently, studies employing AMPs have gained momentum mainly due to the antimicrobial and antibiofilm activity along with their immunomodulatory properties (de la Fuente-Núñez et al., 2012, 2016; Yazici et al., 2018; Haney et al., 2019). EAEC causes chronic as well as persistent diarrhea that eventually damage the intestinal epithelium in human infants and young animals (Lima et al., 2018).

In the present study, Indolicidin, was explored for its antimicrobial activity against MDR-EAEC. Short-chain (12–50 amino acid) AMPs with cationic amino acids and high proportion of hydrophobic residues (∼50%) were reported to be effective against bacterial pathogens (Zasloff, 2002). The tryptophan residues of Indolicidin (39%), due to its preference to the interfacial regions of lipid bilayers, is suggested to disrupt bacterial cytoplasmic membrane by channel formation, thereby inhibiting DNA replication resulting in bacterial filamentation (Falla et al., 1996; Chan et al., 2006). Indolicidin tested in this study could withstand high-end temperatures that are involved in the processing of food ingredients while, the instability of Indolicidin with trypsin could be attributed by the fact that the AMPs with cationic nature exhibit faster degradation. Finally, the stability of Indolicidin against physiological concentration of cationic salts was mainly attributed to its amino acid composition wherein, the peptides with tryptophan and arginine residues were known to improve their antimicrobial activity under challenging salt conditions (Mohamed et al., 2016).

Indolicidin exhibited marginal hemolysis in sheep RBCs; however, the results need to be extrapolated to other cytotoxicity assays before its therapeutic utility. In the present study, Indolicidin decreased the viability of HEp-2 and RAW 264.7 cells in a concentration-dependent manner Supplementary Figure S1. Moreover, the typical characteristics of cytotoxic effect like, a detachment of confluent cell monolayer, vacuolization of the cytoplasm were remarkably observed at higher (4X MIC) concentration might be ascribed to the mechanism of action of the AMPs, but the exact mechanism by which cytotoxicity differed was not yet completely comprehended (Vaucher et al., 2010). Regardless of Indolicidin treatment, a non-significant effect observed on the tested *L. acidophilus* and *L. rhamnosus* Supplementary Figure S5 which reiterated the fact that AMPs are evolutionarily conserved effector molecules of the innate immune system within the gut and are considered safe for commensal gut flora (Ageitos et al., 2017).

The membrane damage exhibited by Indolicidin as evidenced by flow cytometry Supplementary Figure S2 could be due to their optimum hydrophobicity along with the membrane-bound pore formation that would eventually lead to the membrane lipid-bilayer partition (Kumar et al., 2018). Indolicidin is proposed to initiate bactericidal activity through such dissipation of membrane potential without permeabilizing cytoplasmic membrane Supplementary Figures S3, S4 to small molecules (Romani et al., 2013). Indolicidin exhibited a complete elimination of MDR-EAEC in time-kill kinetic assay by 2 h pi while, meropenem exhibited similar inhibition after 60 min. This bacterial clearance by AMPs represents a unique advantage over conventional antibiotics for better treatment outcomes. Increasing positively charged residues would be beneficial for initial electrostatic interactions between AMPs and negatively charged bacterial membrane components and thereby imposing selectivity (Zhang et al., 2016). It could also be inferred that the position of positively charged residues in the AMPs could significantly influence the antimicrobial activity; hence, a clear correlation could be established between the net charge and the antimicrobial activity of AMPs as reported earlier (Romani et al., 2013; Zhang et al., 2016; Kim et al., 2018; Kumar et al., 2018). Similarly, membrane compromising effects were also noticed in MDR-pathogens namely, *Acinetobacter baumannii*, *Pseudomonas aeruginosa*, MRSA, *Staphylococcus pseudintermedius*, *Candida albicans*, when treated with AMPs viz., HD5, Hp1404, LI-F type peptides, RRIKA, PuroA, respectively (Shagaghi et al., 2017; Kim et al., 2018).

Insects and mammals share common mechanisms in their cellular and humoral innate immune response to the pathogens (Tsai et al., 2016; Wojda, 2017). This unique feature enabled *G. mellonella* to be chosen as an alternative host model for investigating the efficacy of AMPs on MDR-EAEC. Though earlier researchers have documented the effect of antimicrobial agents on *G. mellonella* larvae on MRSA, *P. aeruginosa*, *Klebsiella pneumoniae*, Carbapenem-resistant Enterobacteriaceae (Kim et al., 2018), the present study appears to be the first of its kind to explore the effect of AMPs against MDR-EAEC strains on *G. mellonella* larval model. In this study, a dose-dependent lethality of MDR-EAEC strains on the survival of *G. mellonella* larvae was observed Supplementary Figure S6 wherein, the survival was reduced with an increasing MDR-EAEC inoculum concentration. We observed significant survival rate of meropenem-treated larvae, as reported earlier (Benthall et al., 2015), which might either be due to diverse pharmacokinetic parameters as compared to the humans, with a better bioavailability of antibiotics in larvae. Moreover, when the MDR-EAEC infected larvae were treated with Indolicidin, a significant increase in the survival rate was observed as reported earlier by using different antimicrobial agents against MRSA, *A. baumannii*, *Fransicella tularensis*, *Burkholderia multivorans* (Brackman et al., 2011; Zhang et al., 2016). Although complete survival and lack of melanization of uninfected larval groups could be suggestive of *in vivo* safety of AMPs, the obtained data could be extrapolated in the light of *in vitro* cytotoxicity assay using cell lines and LDH cytotoxicity assay performed using larval hemolymph. Our findings suggested that Indolicidin has got an equal or even better efficacy than meropenem, even though the exact peptide-host interaction remained unclear.

The hemolymph burden serves as an indicator in measuring the microbial burden of larvae performed routinely by direct plating and enumeration of microbes for exploring the infection dynamics (Brackman et al., 2011). MDR-EAEC counts reduced significantly over 24 and 48 h pi, possibly be due to its bactericidal effect and/or intermediates produced in the process of melanization (Brackman et al., 2011). The clearance of MDR-EAEC at 96 h pi could be attained either by hemocyte-mediated aggregation and nodulation or phagocytosis of the EAEC strains, resulting in the secretion of larval AMPs, hemocyte cell death and further melanization of the larvae (Brackman et al., 2011).

Multi-drug resistant enteroaggregative *Escherichia coli* stimulated hemocytes might have successfully phagocytosed the pathogen during the early stages of infection (6–18 h pi) that correlated well with the results of bacterial enumeration assay, wherein, significant differences in the MDR-EAEC counts were not observed between infected control group as well as infected larval groups treated with Indolicidin and meropenem. A decline in the circulating hemocytes observed in all the groups at 72 and 96 h pi could probably be a consequence of the bacterial cytotoxic activity on the host cells (Mukherjee et al., 2010). This depletion in hemocyte density might also be attributed to the death of infected hemocytes and/or sequestration of hemocytes in the nodules (Mukherjee et al., 2010; Lu et al., 2014). In order to quantify this innate immune response, the melanization was assessed as a level of PO activity.

The findings of melanization assay correlated well with hemocyte enumeration assay, with decreasing hemocyte density. The elevated melanization intensity (12–48 h pi) observed in treatment groups infected with MDR-EAEC could be correlated with the triggering of PO melanization cascade by activating hemocytes which in turn lead to AMP secretion in the insect fat body, analogous to mammalian liver (Lu et al., 2014). While correlating the findings, it was imperative that Indolicidin improved immunomodulatory effect in the larvae, as the melanization intensity was retained up to 96 h pi in both uninfected and infected larval groups treated with AMP. Similar immunomodulation employing AMPs was observed in mammalian system, suggesting the potential of AMPs as ideal candidates for future drug development (Kumar et al., 2018). A similar trend of increase in LDH production was observed in meropenem-treated group, as reported in an earlier study, wherein ampicillin was employed for the treatment of *P. aeruginosa* in *G. mellonella* larvae (Benthall et al., 2015).

Histopathological examination of whole larvae was necessitated in order to decipher the chronological events related to host-pathogen interaction, hemocyte recruitment and migration of pathogen to different sites (Lu et al., 2014). In the infection control group, the hemocyte-mediated phagocytosis occurred at a rapid pace with the hemocyte recruitment directed toward heart region, where they bind to cardiac muscle and continue phagocytosing microbes during 24 and 48 h pi. A decrease in the bacterial load, melanization rate and the number of circulating hemocytes observed at 72 h pi might be related to the hemocyte recruitment in the heart region and adjoining organs (pericardial cells, fat body), with an attempt to eliminate the pathogen. The evoked immune response that recruited hemocytes in the heart region and adjoining organs in an attempt to eliminate the pathogen could explain the scanty distribution of hemocytes at 72 h pi Interestingly, it was observed that the histopathological examination of larval model correlated with the estimation of bacterial burden and immune markers along with the *in vitro* time-dependent growth kinetics. Besides, it could be well inferred that those bacterial factors that enable survival within the insect host are most likely to be directly relevant to human infection (Tsai et al., 2016).

## Conclusion

We investigated the antimicrobial efficacy of Indolicidin against MDR-EAEC strains in *G. mellonella* larval model for the first time. Indolicidin was found to be stable at high-end temperatures, proteinase-K and physiological concentration of cationic salts; proved to be safe to eukaryotic cells and commensal gut flora; further, exhibited complete elimination of MDR-EAEC. Moreover, significant difference in the MDR-EAEC counts were observed in Indolicidin treated groups as compared to the infected control at 24 and 48 h pi. Further, Indolicidin exhibited an increased immunomodulatory effect evidenced from melanization assay and hemocyte enumeration and proved to be non-cytotoxic to the larval cells by LDH cytotoxicity assay. Indolicidin was found to be efficacious in *G. mellonella* larvae. It provided scope for avenues for testing in ethically less desirable mammalian models (mice/piglets) and also using targeted drug-delivery systems.

## Data Availability Statement

The raw data supporting the conclusions of this manuscript will be made available by the authors, without undue reservation, to any qualified researcher.

## Author Contributions

DR, SM, and SB contributed to the conception and design of the study. JV and RP organized the experiments. MK and SR performed the statistical analysis. JV and MK wrote the first draft of the manuscript. JV, RP, MK, NK, and SR wrote the sections of the manuscript. DR, SM, NK, and SB edited the manuscript. All authors contributed to the manuscript revision, read, and approved the submitted version.

## Conflict of Interest

The authors declare that the research was conducted in the absence of any commercial or financial relationships that could be construed as a potential conflict of interest.
