# Supplementary material for: Antimicrobial Efficacy of Indolicidin Against Multi-Drug Resistant Enteroaggregative Escherichia coli in a Galleria mellonella Model
Source: Front Microbiol. 2019 Nov 29;10:2723. doi: 10.3389/fmicb.2019.02723 (PMC6895141; doi:10.3389/fmicb.2019.02723)
Supplement: Supplementary file 1 [file Data_Sheet_1.doc]

**Table S1.** **Physico-chemical properties of Indolicidin**

| **Peptide designation** | **Amino acid sequence** | **Length** | **Class** | **Molecular weight** | **Extinction coefficient** | **Hydrophobic residue (%)** | **Net Charge (at pH 7.0)** | **Isoelectric point** | **Mechanism of action** |
| --- | --- | --- | --- | --- | --- | --- | --- | --- | --- |
| Indolicidin | ILPWKWPWWPWRR | 13 | Extended | 1906.035 | 27500 | 53.0 | 2.976 | 12.517 | Inhibits DNA synthesis; Outer membrane permeabilisation |

**Table S2.** ***In vitro* antibiotic susceptibility testing of MDR-EAEC isolates**

| **NCBI GenBank Accession No.** | **Antibiotic Susceptibility testing** | | | | | | | | | | **MIC Values (µM)** | | | | | | | | |
| --- | --- | --- | --- | --- | --- | --- | --- | --- | --- | --- | --- | --- | --- | --- | --- | --- | --- | --- | --- |
|  | **CIP** | **CTR** | **AMP** | **COT** | **TE** | **IMI** | **SXT** | **GEN** | **CL** | **CO** | **CIP** | **CTR** | **AMP** | **COT** | **TE** | **IMI** | **GEN** | **CL** | **CO** |
| **KY941936.1 (MDR 1)** | S | S | R | R | R | S | R | S | S | S | S | S | >240 | >240 | 60 | S | S | S | S |
| **KY941937.1**  **(MDR 2)** | R | R | R | R | R | S | S | S | S | S | 120 | >240 | >240 | >240 | 2 | S | S | S | S |
| **KY941938.1**  **(MDR 3)** | R | R | R | R | R | S | R | S | S | S | 60 | >240 | >240 | >240 | 30 | S | S | S | S |

CIP- Ciprofloxacin, CTR- Ceftriaxone, AMP- Ampicillin, COT- Co-trimoxazole, TE- Tetracycline, IMI- Imipenem, SXT- Sulfamethoxazole, GEN- Gentamicin, CL- Chloramphenicol, CO- Colistin sulphate, S – Sensitive, R- Resistant.

**S1. Determination of Minimum Inhibitory Concentration (MIC) and Minimum Bactericidal Concentration (MBC)**

The MIC and MBC values of Indolicidin against ‘planktonic’ MDR-EAEC cells were determined using micro-broth dilution method [1]. The lowest dilutions of AMP revealing no visible growth by naked eye were designated as MIC, while, MBC value was determined at the end of 24 h incubation period. Approximately 10 µL of seeded inoculum was drawn from each well having no visible growth and were placed on Eosin Methylene Blue agar (HiMedia, Mumbai, India). The lowest concentration that produced at least 99.9% killing of the initial inoculum of planktonic cells was considered as the MBC [2].

The MBC values for Indolicidin were either equal or two-fold greater than the MIC values (Table 1) against MDR-EAEC strains. The MIC values for the antibiotics (ampicillin, ceftazidime and ciprofloxacin) used against *E. coli* ATCC 25922 were within the prescribed CLSI range (data not shown).

**S2*. In vitro* stability assays of Indolicidin**

Indolicidin was individually explored for *in vitro* stability assays by subjecting to varied temperatures, proteases as well as physiological concentration of salts.

**S2*.*1 Effect of temperature**

The effect of temperature on the antimicrobial activity of Indolicidin was investigated by incubating the AMP at 70^0^C and 90^0^C for 5, 15 and 30 min and measuring the MIC value on 96-well microtiter plate [3]. An untreated AMP kept at room temperature, was used as a control for each time interval.

Indolicidin was found to be thermostable as evidenced by retained antimicrobial activity after incubation at 70^0^C and 90^0^C for 5, 15 and 30 min, respectively (Table S3).

**Table S3. *In vitro* thermostability observed for Indolicidin** against MDR-EAEC isolates

| **Temperature**  **90^0^C** | **Incubation Time**  **(min)** | **Indolicidin** | | |
| --- | --- | --- | --- | --- |
|  |  | **MDR 1** | **MDR 2** | **MDR 3** |
| **MIC (µM)** | **5** | 32.0 | 32.0 | 32.0 |
|  | **15** | 32.0 | 32.0 | 32.0 |
|  | **30** | 32.0 | 32.0 | 32.0 |
| **MBC (µM)** | **5** | 64.0 | 64.0 | 32.0 |
|  | **15** | 64.0 | 64.0 | 32.0 |
|  | **30** | 64.0 | 64.0 | 32.0 |

| **Temperature**  **70^0^C** | **Incubation Time**  **(min)** | **Indolicidin** | | |
| --- | --- | --- | --- | --- |
|  |  | **MDR 1** | **MDR 2** | **MDR 3** |
| **MIC (µM)** | **5** | 32.0 | 32.0 | 32.0 |
|  | **15** | 32.0 | 32.0 | 32.0 |
|  | **30** | 32.0 | 32.0 | 32.0 |
| **MBC (µM)** | **5** | 64.0 | 64.0 | 32.0 |
|  | **15** | 64.0 | 64.0 | 32.0 |
|  | **30** | 64.0 | 64.0 | 32.0 |

**S2*.*2 Effect of proteases**

The effect of proteases (trypsin and proteinase-K) on the antimicrobial activity of Indolicidin [Protease: AMP- 1:100 (w/w)] was investigated by incubating with the respective protease at 37^0^C for 30 s, 2, 5, 15 and 30 min [3]. Subsequent to the incubation, protease activity was inactivated by heating the samples at 90^0^ C for 10 min and the antimicrobial activity was then determined by measuring the MIC value.

While treating Indolicidin with trypsin, the antimicrobial activity (MIC) remained the same upto 2 min, thereafter, a two-fold decrease was observed. However, the antibacterial activity (MBC) of Indolicidin remained the same throughout the incubation period (Table S4A). On exposure to proteinase-K, the antimicrobial activity (MIC and MBC) of Indolicidin remained the same (Table S4B). Indolicidin tested was found to be protease stable, except for trypsin exposure.

**Table S4A. *In vitro* protease (trypsin) stability observed for Indolicidin against MDR-EAEC isolates**

| **TRYPSIN** | **Incubation Time**  **(min)** | **Indolicidin** | | |
| --- | --- | --- | --- | --- |
|  |  | **MDR 1** | **MDR 2** | **MDR 3** |
| **MIC (µM)** | **0.5** | 32.0 | 32.0 | 32.0 |
|  | **2** | 32.0 | 32.0 | 32.0 |
|  | **5** | 64.0 | 64.0 | 64.0 |
|  | **15** | 64.0 | 64.0 | 64.0 |
|  | **30** | 64.0 | 64.0 | 64.0 |
| **MBC (µM)** | **0.5** | 64.0 | 32.0 | 32.0 |
|  | **2** | 64.0 | 32.0 | 32.0 |
|  | **5** | 64.0 | 64.0 | 64.0 |
|  | **15** | 64.0 | 64.0 | 64.0 |
|  | **30** | 64.0 | 64.0 | 64.0 |

**Table S4B. *In vitro* protease (Proteinase-K) stability observed for Indolicidin against MDR-EAEC isolates**

| **PROTEINASE-K** | **Incubation Time**  **(min)** | **Indolicidin** | | |
| --- | --- | --- | --- | --- |
|  |  | **MDR 1** | **MDR 2** | **MDR 3** |
| **MIC (µM)** | **0.5** | 32.0 | 32.0 | 32.0 |
|  | **2** | 32.0 | 32.0 | 32.0 |
|  | **5** | 32.0 | 32.0 | 32.0 |
|  | **15** | 32.0 | 32.0 | 32.0 |
|  | **30** | 64.0 | 32.0 | 32.0 |
| **MBC (µM)** | **0.5** | 64.0 | 32.0 | 32.0 |
|  | **2** | 64.0 | 32.0 | 32.0 |
|  | **5** | 64.0 | 32.0 | 32.0 |
|  | **15** | 64.0 | 32.0 | 32.0 |
|  | **30** | 64.0 | 32.0 | 32.0 |

**S2*.*3 Effect of physiological concentration of salts**

The stability of Indolicidin in presence of high salt concentrations, the MICs of AMPs was estimated in Cation adjusted- Mueller Hinton (CA-MH) broth with added concentrations of NaCl (150 mM) and MgCl_2_ (2 mM) and in regular MH broth [4]. Indolicidin tested retained their antimicrobial activity during incubation at higher concentrations of salts (150mM NaCl and 2mM MgCl_2_) (Table S5).

**Table S5. *In vitro* salt stability observed for Indolicidin against MDR-EAEC isolates**

| **NaCl**  **(150 mM)** | **Indolicidin** | | |
| --- | --- | --- | --- |
|  | **MDR 1** | **MDR 2** | **MDR 3** |
| **MIC (µM)** | 32.0 | 32.0 | 32.0 |
| **MBC (µM)** | 64.0 | 64.0 | 32.0 |

| **MgCl_2_**  **(2 mM)** | **Indolicidin** | | |
| --- | --- | --- | --- |
|  | **MDR 1** | **MDR 2** | **MDR 3** |
| **MIC (µM)** | 32.0 | 32.0 | 32.0 |
| **MBC (µM)** | 64.0 | 64.0 | 32.0 |

**S3*. In vitro* safety assays of Indolicidin**

The safety of Indolicidin on host cells was determined using haemolytic assay using sheep erythrocytes and cytotoxicity assay using cell lines.

**S3*.*1 Haemolytic assay**

The haemolytic activity of Indolicidin was tested by determining the extent of haemoglobin release from erythrocyte suspension of fresh sheep blood cells [10% (v/v) in Tris buffer, pH 7.40] [3]. Indolicidin was dissolved in Tris buffer (100 μL) to form 1X, 2X and 4X MIC and was added to the resuspended blood cells (100 μL) in 96-well plates. The buffer solution and 0.1% Triton X-100 were used as the negative and positive controls, respectively. The haemoglobin release was monitored by measuring the absorbance at 540 nm (Thermo Scientific Multiskan GO) and the haemolysis at each AMP concentration was estimated as, Haemolysis (%) = 100 x (A_exp_ – A_Tris_)/ (A_TritonX-100_ – A_Tris_) wherein, A_exp_ is the experimental absorbance; A_Tris_ is the absorbance of negative control and A_TritonX-100_ is the absorbance of positive control.

Indolicidin tested was found to be non-haemolytic at 1X and 2X MIC concentrations; however, a minimal haemolysis (<5%) was noticed at 4X MIC for the AMP tested (Table S6).

**Table S6. *In vitro* cytotoxicity of Indolicidin (at different MIC concentrations) against sheep RBCs**

| **Concentration of Indolicidin** | **Haemolysis (%)** |
| --- | --- |
| **MIC (1X)** | 0.0 |
| **MIC (2X)** | 0.0 |
| **MIC (4X)** | 4.49 |

**S3*.*2 Cytotoxicity assay**

The *in vitro* effect of Indolicidin on the viability of human epithelioma cell line, HEp-2 and murine macrophage cell line, RAW 264.7 was evaluated using the MTT [3-(4,5-dimethylthiazole-2-yl)-2,5-diphenyl tetrazolium bromide] assay [5]. The pre-cultured adherent cells in DMEM (pH 7.20) were transferred to a 96-well plate (1x10^5^ cells/well) to form monolayers which were treated with 200 µL of Indolicidin (1X, 2X and 4X MIC) diluted in DMEM and maintained for 24 h at 37^0^C in a humidified incubator with 5% CO_2_. Cells incubated with fresh DMEM served as negative control; supernatant was then removed, proceeded further using MTT assay kit (Abcam, USA) and the cytotoxicity was monitored by measuring the absorbance at 590 nm. The percentage of cytotoxicity was calculated as, 100 x (Control– Sample)/ (Control) wherein, control is the absorbance of the untreated cell control and sample is the absorbance of AMP treated cell. Additionally, the cells treated with Indolicidin were monitored using an inverted optical microscope (Nikon, Japan) to determine the effects on cell morphology.

The addition of Indolicidin marginally decreased the cell viability of human epithelioma cell line (HEp-2) and murine macrophage cell line (RAW 264.7), in a concentration-dependent manner (Fig. S1). The most typical morphological changes caused by the peptide were shrinkage and vacuolisation of cytoplasm and loss of monolayer at 4X MIC concentrations. Cells treated with lower peptide concentrations (1X and 2X) did not exhibit remarkable morphological changes (data not shown).


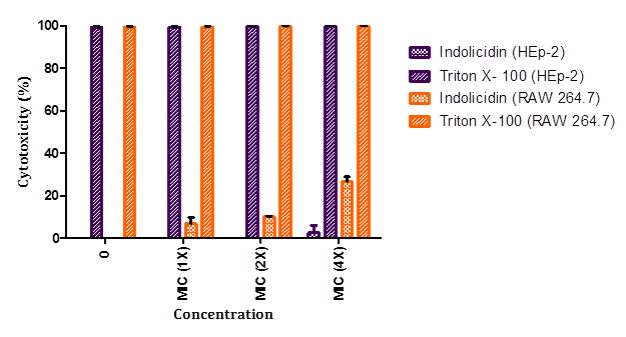


**Fig. S1.** ***In vitro* cytotoxicity observed for Indolicidin at different concentrations on HEp-2 and RAW 264.7 cells.**

**S4. *In vitro* membrane permeability assay of Indolicidin**

The membrane permeability effect of Indolicidin on MDR-EAEC was probed by incubating the AMP-treated bacterial cells with the membrane impermeant dye, Propidium Iodide (PI) followed by flow cytometry (FACS) analysis to determine AMP-induced membrane damage [7]. The staining of nucleic acids in cells with PI indicates compromised cell membrane structure and cell death; hence, PI uptake versus exclusion can be used to discriminate dead cells, in which plasma membranes become permeable, regardless of the mechanism of death, from live cells with intact membranes. In brief, MDR-EAEC cell suspension (ca. 1x10^7^ CFU/ml) harvested and resuspended in PBS (50 µl) was treated with 1X MIC concentrations (50 µl) of Indolicidin. The untreated MDR-EAEC served as negative control and the meropenem-treated MDR-EAEC cells were used as positive control. The bacterial mixture, after incubation at 37^0^C for 30 min were resuspended in Flow Cytometry Staining Buffer (500 µl). Further, in order to adjust flow cytometer settings for PI, add 10 µl of PI staining solution (final concentration of 50 µg/ml) to a control tube of otherwise unstained cells and mixed gently. The fluorescence of PI with a laser excitation of 488 nm was determined using the FL-2 channel (BD FACSCalibur, USA); the scatter data for unstained cells and single-color positive controls was acquired using Cell Quest Pro software. The stop count was set on the viable cells from a dot-plot of forward scatter versus PI and the histogram of unstained bacterial control, meropenem-treated and the peptide(s)-treated MDR–EAEC were retrieved with the FL-2 H channel values on X-axis against the counts plotted on Y-axis.

Indolicidin showed a remarkable potential to damage the cell membrane of MDR-EAEC strains (PI-positive cells >50%) (Fig. S2).

**
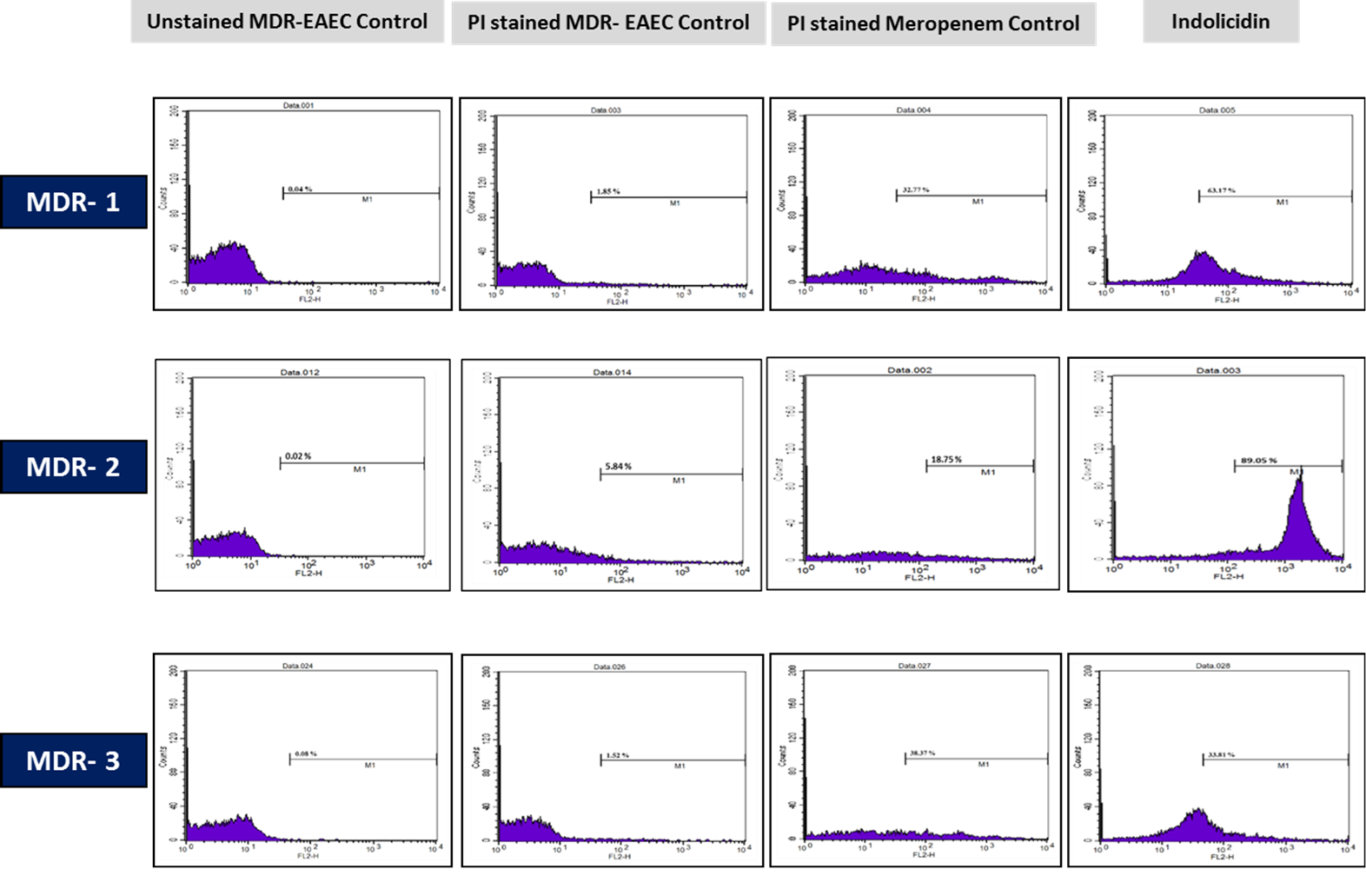
**

**Fig. S2.** ***In vitro* membrane permeability of Indolicidin against MDR-EAEC strains by flow cytometry.** Histograms reveal PI uptake by MDR-EAEC on exposure to Indolicidin with appropriate control groups; rows represent three MDR-EAEC strains tested while, columns denote the bacterial cells exposed to meropenem and Indolicidin.

**S5. *In vitro* inner membrane permeability assay of Indolicidin by ONPG**

The inner membrane permeability of Indolicidin was assessed by ONPG [8]. Sterile PBS served as negative control whereas, Meropenem was used as the positive control. The absorbance was read at 420 nm for 120 min, with the readings taken at 5 min interval at 37^0^C in ELISA microplate reader (Thermo Scientific Multiskan GO) with shaking. The variation of the inner membrane permeability (OD) was calculated as, OD_420_ = OD_t_ – OD_0_ wherein, OD_t_ is the value of the AMP-treated sample and OD_0_ is the PBS treated control.

The inner membrane permeabilisation was not prominent with Indolicidin, even at MBC concentrations (Fig. S3).


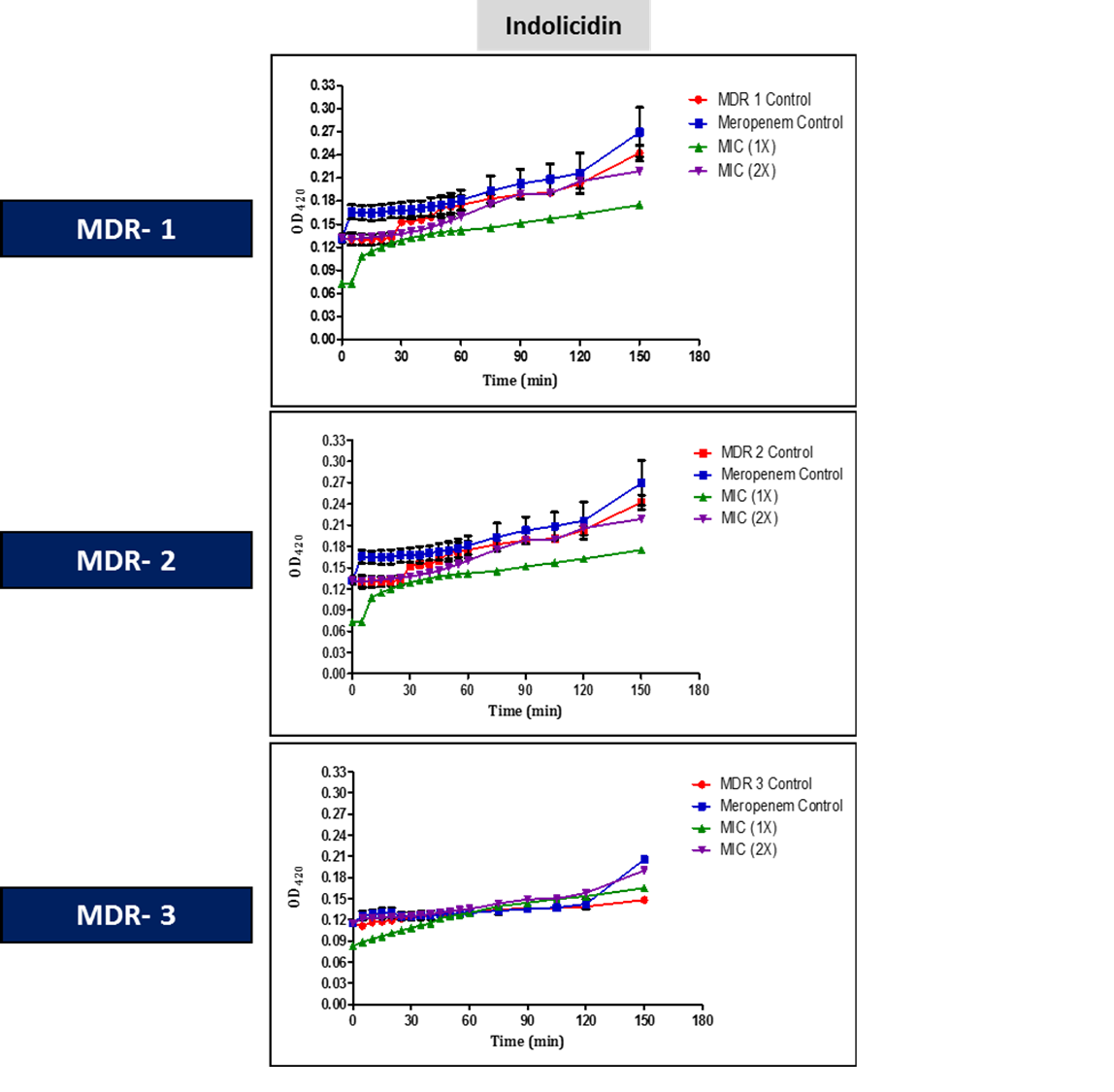


**Fig.** **S3. *In vitro* inner membrane permeability assay of MIC (1X and 2X) concentrations of Indolicidin against MDR-EAEC strains employing ONPG expressed as a function of time at 37^0^C.** Hydrolysis of ONPG by β-galactosidase was used to monitor inner membrane permeabilisation by absorbance at 420 nm.

**S6. *In vitro* outer membrane permeability assay of AMPs by nitrocefin**

The outer membrane permeability of Indolicidin was assessed by nitrocefin [9]. Sterile PBS served as negative control whereas, Meropenem was used as the positive control. The absorbance was read at 486 nm for 120 min, with the readings taken at 5 min interval at 37^0^C in ELISA microplate reader, with shaking. The variation of the outer membrane permeability (OD) was calculated as, OD_486_ = OD_t_ – OD_0_ wherein, OD_t_ is the value of the AMP- treated sample and OD_0_ is the PBS treated control.

Indolicidin permeabilised the outer membrane of the MDR-EAEC strains in a concentration and time-dependent manner (Fig. S4). Also, the onset and progress of the permeabilisation by Indolicidin was much rapid than the positive meropenem control. Moreover, the concentration of nitrocefin permeation for the AMP-treated strains was much higher than the meropenem-treated strains.

**
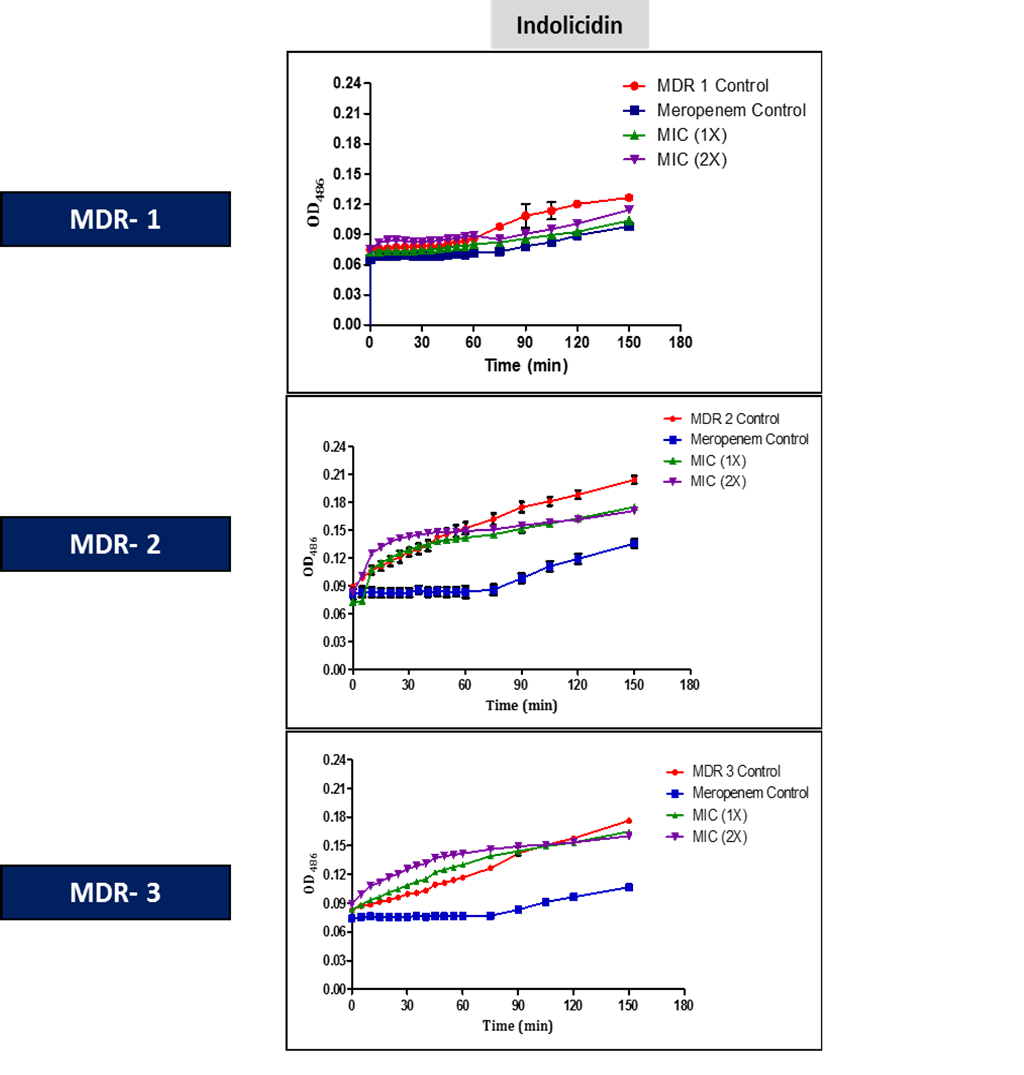
**

**Fig. S4.** ***In vitro* outer membrane permeability assay of MIC (1X and 2X) concentrations of Indolicidin against MDR-EAEC strains employing nitrocefin expressed as a function of time at 37^0^C.** Hydrolysis of nitrocefin by β-lactamase was used to monitor outer membrane permeabilisation by absorbance at 486 nm.

**S7. *In vitro* antibacterial effect of Indolicidin on commensal gut flora**

Briefly, Mann Rogosa Sharpe (MRS) broth medium (100 µl) containing Indolicidin (1X MIC) was inoculated with 100 µl of each commensal bacteria (ca. 1x10^7^ CFU/ml) in 96-well microtiter plates. Each plate included a positive growth control (untreated commensal flora) and a negative control (MRS broth). Subsequent to the incubation at 37^0^C for 48 h, Indolicidin was tested for the effect on commensal flora by observing the absorbance at 600 nm (Thermo Scientific Multiskan GO) as well as drawing two 10 µl samples from each well and plating the samples onto MRS agar plates [6].

The commensal bacteria (*L. acidophilus* and *L. rhamnosus*) revealed similar growth pattern, regardless of AMP treatment (Fig. S5). Overall, a non-significant (*P*> 0.05) antimicrobial effect was observed for Indolicidin tested against *L. acidophilus* and *L. rhamnosus*, suggesting its safety against commensal bacteria.


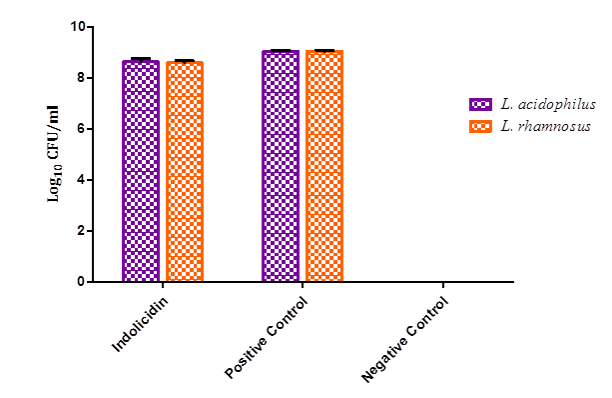


**Fig. S5.** ***In vitro* effect of Indolicidin on commensal gut flora.** Untreated *L. acidophilus* and *L. rhamnosus* serve as positive growth control while, media (MRS broth) serve as negative control.

**S8. *In vitro* dose- and time-dependent growth kinetics of MDR-EAEC with Indolicidin**

The log-phase bacterial inoculum was enumerated using McFarland standard tubes and also by plate count method using eosin-methylene blue (EMB) agar containing 100 µg of ampicillin (HiMedia Laboratories, Mumbai, India). The desired bacterial numbers for each MDR-EAEC isolate and Indolicidin were suspended in CA-MH broth as follows: Group I, 10^7^ CFU of MDR- EAEC (50 µl) with 1X MIC Indolicidin (50 µl); Group II, 10^7^ CFU of MDR- EAEC (50 µl) with MBC Indolicidin (50 µl); Group III, 10^7^ CFU of MDR- EAEC (50 µl) with Meropenem (10 µg/ml; 50 µl); Group IV, 10^7^ CFU of MDR- EAEC (50 µl) with CA-MH broth (50 µl); Group V, 10^7^ CFU of MDR- EAEC (50 µL) with 4X MIC Indolicidin (50 µL). Similar groups were also made for the other two MDR-EAEC isolates. The respective groups along with the appropriate controls were incubated at 37^0^C up to 72 h.

**S9. Determination of LD_50_ dose of MDR-EAEC strains**

Different doses (1x10^4^; 1x10^5^; 1x10^6^; 1x10^7^; 1x10^8^; 1x10^9^; 1x10^10^ and 1x10^12^ CFU) of each MDR-EAEC test isolates were inoculated separately into *G. mellonella* larvae (n = 9 per strain per dose). Normally, within 24 h of infection, the larvae exhibit progressive melanisation and death, corresponding to the virulence potential of MDR-EAEC infection. The survival rates of the infected larva were monitored at every 3 h interval upto 120 h post-infection (pi) and the observations were plotted using Kaplan-Meier method (Fig. S6). The dose of MDR-EAEC strains representing death in half of the infected larvae was designated as LD_50_; the validated LD_50_ dose was used for further *in vivo* studies to evaluate the antibacterial effect of AMPs.


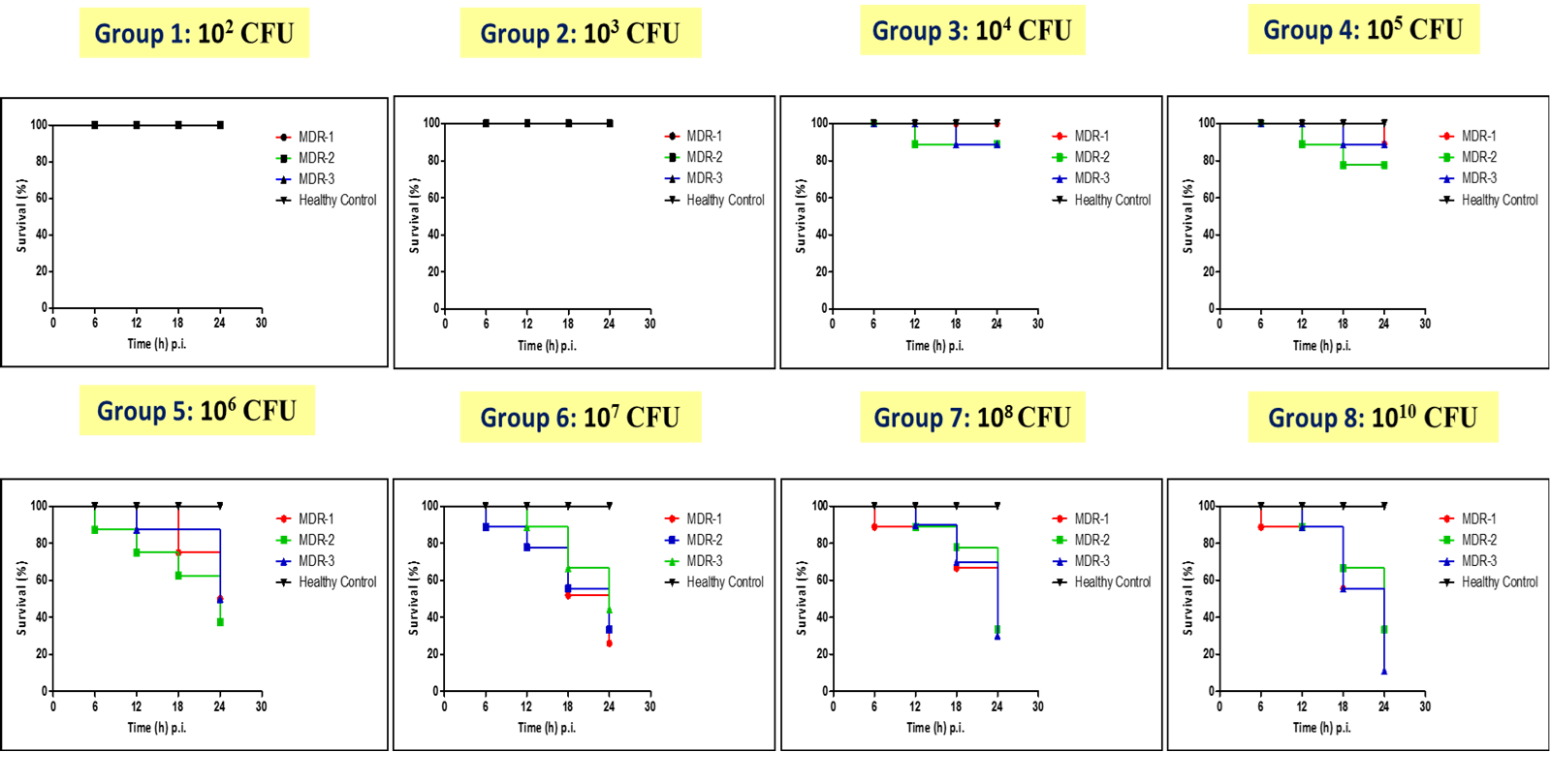


**Fig. S6.** **MDR-EAEC infection of *Galleria mellonella* larvae.** Larvae were infected with 3 different MDR-EAEC strains and injected with 8 different doses of bacteria. All results represent means of at least 3 independent experiments with 9 larvae per treatment. Survival curves were plotted using Kaplan-Meier method and statistical analysis were performed using the log rank test for multiple comparisons (GraphPad Software, San Diego, CA).

**S10. Melanisation assay**

For determination of the melanin production, an immune marker, the *G. mellonella* (n=3 larvae per group) were investigated at an interval of 6 h pi for 24 h, followed by 24 h interval till 96 h [10]. An aliquot of the pooled haemolymph (100 µl) was transferred into 96-well microtiter plate for measuring the optical density (OD) at a wavelength of 450 nm using ELISA plate reader (Thermo Scientific Multiskan GO) with OD values of haemolymph collected from apparently healthy un-inoculated larvae as the background control.

**S11. Enumeration of MDR-EAEC counts**

The haemolymph of *G. mellonella* (n=3 larvae per group) were collected aseptically at an interval of 6 h pi upto 24 h, followed by 24 h interval till 96 h pi in sterile NSS. The haemolymph was vortexed thoroughly and serially diluted 10-fold in sterile NSS and the bacterial burden was assessed on EMB agar plates supplemented with 100 µg of ampicillin per plate [6]. The number of MDR-EAEC colonies on the plates was counted and expressed as log_10_CFU/ml of haemolymph.

**S12.LDH cytotoxicity assay**

The positive and negative controls used in this study were distilled water and 20% Triton X-100, respectively. The absorbance was read using ELISA plate reader at 500 nm; the haemolymph from untreated larvae served as background control and the cytotoxicity was calculated as, Cytotoxicity (%) = (OD_Sample_ - OD_Control_)/ (OD_Total Lysis_ - OD_Control_) x 100, wherein, sample is the control absorbance of treated cell; control is the experimental absorbance of the untreated cell control and total lysis is the absorbance of Triton X-100 treated cells.

**S13. Histopathological Examination**

The neutral buffered formalin (10%)-fixed whole larvae were dissected transversally into two halves by means of anatomic pincers and by using a new lancet blade for each larva. The procedure was carefully performed to avoid the squeeze of the larval tissues; each paraffin-embedded sections (3 µm) was then sectioned and stained using Haematoxylin and Eosin (H&E) to evaluate tissue morphology observing standard laboratory protocols (Fig. S7).

As the infection progressed at 24 h pi, a comparatively higher cluster of haemocytes was observed in the sub-cuticular area of group I; fat bodies with points of melanisation and increased load of bacteria were also seen around the tubular organelle (Supporting Information S14). In group II, mild accumulation of bacteria was observed around the organelle with a distribution of scanty haemocytes exhibiting no noticeable aggregates or melanisation. In contrast, groups IV and V looked apparently healthy with individually distributed scanty haemocytes exhibiting no noticeable aggregates or melanisation. Further, at 48 h pi, the clustered haemocytes in sub-cuticular area and fat bodies with an evidence of melanisation and an increased bacterial load around tubular organelle were observed in group I. In group II, scanty distribution of haemocytes with no noticeable aggregates or melanisation were observed with a mild accumulation of bacterial load around the organelle. Surprisingly, all the other larval groups (IV and V) exhibited individually distributed scanty haemocytes with no noticeable aggregates or melanisation. Later, at 72 h pi, a mild accumulation of bacteria was appreciated in group I with a comparative reduction in the melanisation and haemocyte aggregates within the fat bodies while, scanty distribution of haemocytes with no noticeable aggregates or melanisation as well as reduced haemocyte accumulation was noticed in group III. Interestingly, all the other groups (treatment and control) exhibited scanty haemocytes with no noticeable aggregates or melanisation (Fig. S7).


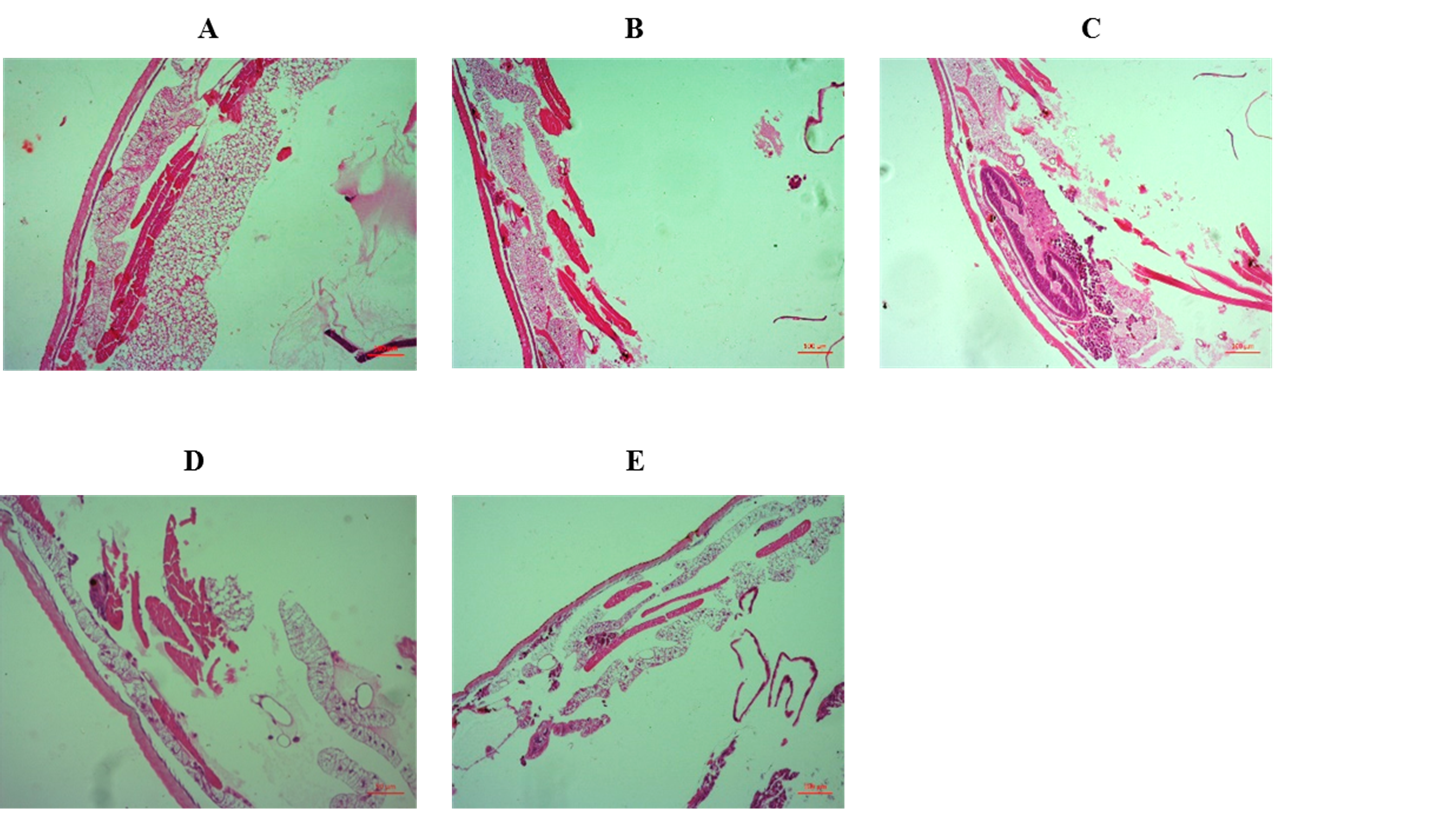


**Fig.** **S7. Histopathological analysis of *G. mellonella* larvae exhibiting the effect of Indolicidin on MDR-EAEC *in vivo* using H&E staining at 24 h pi.** The images denote larvae inoculated with: PBS (A), uninfected larvae treated with Indolicidin (B), LD_50_ dose of MDR-EAEC (C), MDR-EAEC-induced larvae treated with Meropenem 3 h pi (D), MDR-EAEC-induced larvae treated with Indolicidin 3 h pi (E). Representative images are shown from histological analysis of 3 larvae for each condition from 3 independent experiments. Scale bars, 100 µm.

**References**:

1. Clinical and Laboratory Standards Institute (CLSI). Performance Standards for Antimicrobial Susceptibility Testing, 28th ed., M 100, Wayne, USA, 2018.
2. National Committee for Clinical Laboratory Standards (NCCLS). Methods for determining bactericidal activity of antimicrobial agents: Approved guideline. M26-A.Wayne PA, USA, 1999.
3. Ebbensgaard A, Mordhorst H, Overgaard MT, Nielsen CG, Aarestrup FM, Hansen EB. Comparative evaluation of the antimicrobial activity of different antimicrobial peptides against a range of pathogenic bacteria. PLoS. One 2015; 10:e0144611.
4. Mohamed MF, Abdelkhalek A, Seleem MN. Evaluation of short synthetic antimicrobial peptides for treatment of drug-resistant and intracellular *Staphylococcus* *aureus*. Sci Rep 2016; 6:29707.
5. Mosmann T. Rapid colorimetric assay for cellular growth and survival: application to proliferation and cytotoxicity assays. J Immunol Methods. 1983; 65:55-63.
6. Miles AA, Misra SS, Irwin JO. The estimation of the bactericidal power of the blood. Epidemiol Infect 1938; 38(6):732-49.
7. Zhang SK, Song JW, Gong F, Li SB, Chang HY, Xie HM et al. Design of an α-helical antimicrobial peptide with improved cell-selective and potent anti-biofilm activity. Sci Rep 2016; 6:27394.
8. Marri L, Dallai R, Marchini D. The novel antibacterial peptide ceratotoxin A alters permeability of the inner and outer membrane of *Escherichia coli* K-12. Curr Microbiol 1996; 33:40-43.
9. Epand RF, Pollard JE, Wright JO, Savage PB, Epand RM. Depolarization, bacterial membrane composition, and the antimicrobial action of ceragenins. Antimicrob Agents Chemother 2010; 54:3708-13.
10. Wand ME, McCowen JW, Nugent PG, Sutton JM. Complex interactions of *Klebsiella* *pneumoniae* with the host immune system in a *Galleria* *mellonella* infection model. J Med Microbiol 2013; 62:1790-98.
